# An Analysis of the Deleterious Impact of the Infodemic during the COVID-19 Pandemic in Brazil: A Case Study Considering Possible Correlations with Socioeconomic Aspects of Brazilian Demography

**DOI:** 10.3390/ijerph19063208

**Published:** 2022-03-09

**Authors:** Maria da Penha de Andrade Abi Harb, Lena Veiga e Silva, Nandamudi Lankalapalli Vijaykumar, Marcelino Silva da Silva, Carlos Renato Lisboa Francês

**Affiliations:** 1 Institute of Technology, Federal University of Para, Belem 66075-110, Brazil; rfrances@ufpa.br; 2 Center for Exact Sciences and Technology, University of Amazon, Belem 66060-902, Brazil; lena.veiga@unama.br; 3 National Institute for Space Research, Sao Jose dos Campos 12227-010, Brazil; vijay.nl@inpe.br; 4 Institute of Engineering and Geosciences, Federal University of Western Para, Santarem 68040-255, Brazil; marcelino.ss@ufopa.edu.br

**Keywords:** infodemic COVID-19, Google Trends, socio-economic variables, government programs, denialism, clustering, dendrogram

## Abstract

Due to COVID-19, a huge amount of incorrect information has been disseminated on the internet, which may interfere with the disease’s advance. This study analyzes the behavior of the Brazilian population during the pandemic, employing queries of infodemic data searched on Google Trends and relating them to socioeconomic and political indicators in the country. The z-score technique was used to standardize the data; and for multivalued analysis, dendrograms and the Elbow method detected similar patterns among Brazilian states. The result was divided into three analyses. In the analysis of the research trend of infodemic terms, the themes “Prevention and Beliefs” and “Treatment” prevailed. In the exploratory analysis, socioeconomic indicators related to income and education, as well as government programs, showed no impact on infodemic searches; but the results suggest that the states that supported the Brazilian president in the 2018 election, where he obtained more than 50% of the votes, were the states that most searched for infodemic terms: a total of 46.58% more infodemic searches than in the other states. In the multivalued analysis, the socioeconomic indicators used showed similarities in the patterns, highlighting a cluster containing 77% of all Brazilian states. The study concludes that denial about the pandemic and the influence of political leadership can influence infodemic information searches, contributing to a disorganization in the control of disease control and prevention measures.

## 1. Introduction

At the end of the year 2019, China identified a new coronavirus (called SARS-CoV-2) in humans, different from the already known respiratory syndrome coronaviruses. The number of cases increased rapidly and affected other countries, requiring the World Health Organization (WHO) to declare, on March 11, a COVID-19 pandemic, the disease caused by the new coronavirus [1].

Responsible and governmental agencies, such as WHO, the Ministry of Health, and Health Departments, began to disclose information to prevent new sources of contagion and keep the population free from false, malicious, and questionable news, since information from the internet and the media can impair the diagnosis of health and diseases [2], in addition to being difficult to judge information from the internet, especially in the midst of a pandemic [3]. In this context, at the Munich security conference, on 15 February 2020, WHO Director Tedros Adhanom Ghebreyesus said, “we are not just fighting an epidemic, we are fighting an infodemic” [4].

Booklets, images, documents, procedures with guidelines on transmission and protection measures of COVID-19 [5] were made available, digital media being one of the most promising approaches to face the outbreak in modern societies, when information is provided correctly, as seen in Alvarez-Risco et al. [6].

### 1.1. COVID-19 and Infodemic in Brazil

In Brazil, the first case of COVID-19 was reported in the city of São Paulo on 26 February 2020 (less than three months after the first case in China), and since March 2020, many states have adopted isolation and social distancing measures, including the adoption of lockdown measures. The advance of the pandemic occurred differently in the Brazilian states concerning the period of the first wave (2nd quarter of 2020) and second wave (1st quarter of 2021) of contagions. This may have been caused due to the relaxing of isolation measures, combined with the emergence of new variants of SARS-CoV-2, and also because Brazil is a country with large continental dimensions, many social inequalities, and different beliefs/cultures (information on Brazilian states and their regional divisions can be seen in the Supplementary Material S1, Figures S1 and S2).

For Aquino et al. [7] health care resources in Brazil are chronically deficient and unequally distributed and dealing with this aspect is a big challenge, especially in a pandemic. Another challenge is the fact that “Brazilian government authorities exhibit contradictory data regarding the impact of the disease in the country” [8], forms of prevention and treatment, and websites with false or doubtful information.

According to the Digital News Report [9], global concerns about false and misleading information increased in 2021, ranging from 82% in Brazil to just 37% in Germany, for example. This expressive number in Brazil (82%) may be a reaction of the population to the numerous amounts of false or inaccurate information declared by President Jair Bolsonaro, but also linked to sociocultural factors and the way Brazilians perceive science and approach health and denial [10]. President Jair Bolsonaro manifested himself negatively to the pandemic, having 914 false or inaccurate statements in 2020 and 776 statements by June 2021 [11].

According to studies by Rathsam [12] during the COVID-19 pandemic, denial in Brazil took on alarming proportions. President Bolsonaro manifested himself in a contrary and incredulous manner regarding the seriousness of the disease, preventive measures, and underreporting of epidemiological data, in addition to showing a lack of behavior in carrying out national health strategies, by encouraging therapeutic treatments without scientific validation and to discredit the vaccine, among other examples. Thus, the government’s denial accentuates uncertainties and influences the population in relation to adherence to prevention protocols, compromising the fight against the pandemic and threatening democracy.

This context has attracted the attention of researchers who have analyzed the influences and role of authorities in positively or negatively impacting the pandemic [6,13].

### 1.2. Analysis of Google Trends Infodemic

Both the impact of the disease, which was already great, and the lack of information associated with it, allowed disinformation to quickly appear and spread across various social media platforms [14]. For information and clarification, many internet users turned to Google and Google Trends (GT), which is the Google tool that captures the population’s search behavior. Mavragani and Ochoa [15] indicate GT as the most popular tool to address health issues and topics using internet data. Most GT research studies are in health and medicine, focusing primarily on surveillance and analysis of health topics and the prediction of diseases, outbreaks, and epidemics.

Investigations such as in [16,17,18,19,20] use GT data to understand how the population was reacting to COVID-19 issues and also the interest in infodemics, which can change population behavior, thereby impacting actions contrary to the control measures adopted. The analysis of these data can help public authorities with mechanisms to combat a large amount of misinformation that can generate serious social, economic, and environmental consequences.

### 1.3. Rationale

For Rothkopf [21], we should react to infodemics in the same way we react to diseases. Understanding how these ideas are introduced into the population, how they spread, what accelerates their spread, and what their consequences are, is essential to controlling outbreaks and can contain misinformation. This does not mean repressing information. It means effectively managing each outbreak and presenting the facts fully and quickly to critical audiences. In this context, this work, in its analysis, aims to find the factors that impacted searches for infodemic terms in Brazilian states during a year and a half (January 2020 to June 2021) of the pandemic.

We use GT [22] to provide insights and potential indicators of important changes in information-seeking patterns [18,23,24,25] about the COVID-19 pandemic in Brazil. In addition to GT, we use data from Brazilian Government portals.

Based on these data, three analyses related to the infodemic in Brazil are performed:

1. Trend analysis of research on infodemic terms, divided into five themes relating to the pandemic;
2. Spatial Exploratory Analysis of the Brazilian states considering the quantity of researched infodemic terms relating to social and political scenarios in the country;
3. Multivalued analysis based on clustering of Brazilian states considering the quantity of researched infodemic terms, internet access data, numbers of deaths, and COVID-19 cases relating to social and political scenarios in the country.

The scenarios used in the last two analyses are created by comparing the territorial division of Brazil (states) considering the following indicators: social inequality generated from data on income and education; government financial support programs distributed to the underprivileged population; and finally, the result of the 2018 presidential elections to identify the states where President Bolsonaro (denier) has the greatest political support.

Thus, the objective of this study is to analyze the behavior of the Brazilian population during the period of the COVID-19 Pandemic in Brazil in relation to research into infodemic terms, relating it to the country’s socioeconomic and political characteristics.

## 2. Materials and Methods

In this work, a new approach was used to carry out the methodology by combining the database of infodemic terms, extracted from searches in GT, with socioeconomic variables, policies, and information from government income aid programs, in the period of COVID-19 in Brazil. Trend analysis of infodemic terms, exploratory spatial analysis, and finally, multivalued analysis with dendrograms and the Elbow method were used to group the states into similar patterns of behavior.

To carry out the three analyses of the study, we retrieved data from GT [22], and six other free and open access information sources on the internet, with public and nationally consolidated data. Specific data were used in each analysis.

For the study, data were collected from all databases, between 1 January 2020 and 30 June 2021, and grouped by quarters, performing the appropriate treatment in each database. The grouping by quarter was carried out because of the Brazilian Institute of Geography and Statistics (Instituto Brasileiro de Geografia e Estatística, IBGE) [26] database, where the socioeconomic variables used, makes data available by quarter. There are a total of 6 quarters within the capture period, and for better handling of the work, they were called the pandemic quarter (PQ).

### 2.1. Data Extraction

To capture infodemic terms in GT, we used the structure discussed in Mavragani and Ochoa [15]. The authors point to GT as the most popular tool to address health issues and topics using internet data. Data collection was performed on 10 August 2021, capturing normalized search data in the form of relative search volume (RSV) based on the search popularity scale, ranging from 0 (low) to 100 (very popular). We generated national and state level datasets.

The identification of infodemic terms was initially based on the work carried out by Rovetta and Bhagavathula [18,27]. The authors defined infodemic terms as a term, query, or phrase that generates or fuels false news, misinterpretations, or discriminatory phenomena. This work followed this structure, and the first topic researched was for terms on the denomination of the virus, with more generic terms, which could confuse the population due to the lack of specificity. Subsequently, a search was carried out on Brazilian journalistic sites, such as: G1 [28], UOL [29], MSN [30], to identify more infodemic terms, and also more specific infodemic terms in Brazil.

Many terms were searched, including association of terms using the “+”, which works as an “or”. For the work, we used the terms that brought the most significant results and were classified into five themes:

1. Denomination: coronavirus, COVID, corona, SARS;
2. Origin: 5G coronavirus, bill gates + bill gates virus, Chinese virus, China virus;
3. Prevention and Beliefs: no masks, no isolation, gargle, coronavirus gargle, garlic + garlic consumption + eating raw garlic is bad, coronavirus garlic, kill COVID, milk COVID;
4. Treatment: chloroquine, COVID chloroquine, coronavirus chloroquine, chloroquine, chloroquine trump, chloroquine china, ivermectin, how to take ivermectin 6 mg, COVID food;
5. Vaccine: alligator vaccine, Doria vaccine, vaccine kills + DNA vaccine, COVID cancer + COVID cancer vaccine, cause COVID vaccine, alcoholic drink COVID vaccine.

Some terms are very specific for Brazil, such as, ‘alligator vaccine’, which was used due to the great repercussion it had after President Bolsonaro mentioned, in December 2020, that a person who takes the vaccine can turn into an alligator; and also the term ‘Doria vaccine’ was used due to repercussions when the president mentioned that the “vaccine is not from Doria”, or that “the vaccine from Doria has no effect” [31]. João Doria is the current governor of the state of São Paulo (the most populous state in Brazil) and since the beginning of the pandemic, he has been adopting control and prevention measures following WHO guidelines.

Subsequently, the internet access data available on the National Telecommunications Agency website [32] were captured. As internet access structures and technologies are very different between Brazilian regions, these data could help and influence the analysis.

Data from COVID-19 were captured from the Portal of the Coronavirus Panel [33]. We used the spreadsheet that was made available daily, with the new number of cases per day and the number of deaths per day, from all Brazilian cities.

Further data were collected from the IBGE [26]—data from the Continuous National Household Sample Survey (Pesquisa Nacional por Amostra de Domicílios Contínua, PNADC)—which tracks quarterly fluctuations and the evolution, in the short, medium, and long term, of the workforce, and other information necessary for the study of the socioeconomic development of parents. It is a survey carried out by the IBGE in a sample of Brazilian households that investigates several socioeconomic characteristics of society, such as population, education, work, income, housing, social security, migration, fertility, marriage, health, and nutrition, among other topics that are included in the survey according to the information needs of Brazil.

From this base, 20 variables were selected for the study, from a set of more than 200 variables, ranging from the individual’s age and higher education level to whether they had generated income in the previous month and the current month. To extract these data, we used a free environment R (R Foundation for Statistical Computing) with the PNADcIBGE Package [34], which was developed to facilitate the download, import, and analysis of sample data from the PNADC.

In order to understand how government programs could influence, for example, research on the infodemic in Brazil, data from the emergency assistance program of the Transparency Portal [35] were also captured. This program was launched as an emergency to help workers who lost income due to the COVID-19 pandemic and began in April 2020 and ended in October 2021. Also used were data from the Unemployment Insurance, from the Program Portal Dissemination of Labor Statistics [36]. Unemployment Insurance is one of the Social Security benefits and is intended to guarantee temporary financial assistance to workers who are involuntarily dismissed (without just cause), and it has been in operation since 1991.

Political data were extracted from the Superior Electoral Court [37]; those for the 2018 presidential elections were extracted when in the second round two candidates were running for the presidency: Jair Bolsonaro (elected) and his opponent Fernando Haddad.

### 2.2. Data Processing

The pre-processing of data from each database was carried out individually, removing duplicate and missing data, avoiding noise and possible distortions in the veracity of the data, to improve the quality and present more significant results.

All databases have the date field and state field, facilitating the grouping of data by quarters and by state, allowing the appropriate treatments for the fields in these groups, which can be the average or even the sum of the values. Some fields that referred to quantitative information on the population of a state were transformed to the rate of one hundred thousand inhabitants, being proportional to the population size of each state, which can be better interpreted when making comparisons between states with such different populations, such as in Brazil. After this processing, the entire dataset was stored in a single database, containing all quarters.

Table 1 identifies the year and period we used to divide the pandemic quarters. Each state was associated with six PQ, which facilitated the analysis of the research, in terms of follow up and the evolution of population behavior throughout the pandemic.

Some studies for the research were carried out at the national level, covering the entire period. In these cases, there was no division into pandemic quarters. Also, for each analysis, specific data were used, with the multivalued analysis being the base with the largest number of variables.

### 2.3. Data Analysis

Specific data were applied for each analysis studied: in the analysis of trends, the infodemic GT RSVs were used; in the exploratory spatial analysis data on income, education, and infodemic, RSVs (first scenario), followed by data from government programs and infodemic RSVs (second scenario), and, lastly electoral data and infodemic RSVs (third scenario) were used; finally, in multivalued analysis, we used the database with information from the three scenarios, plus information on internet access and numbers of deaths and cases from COVID-19.

**Figure 1:**
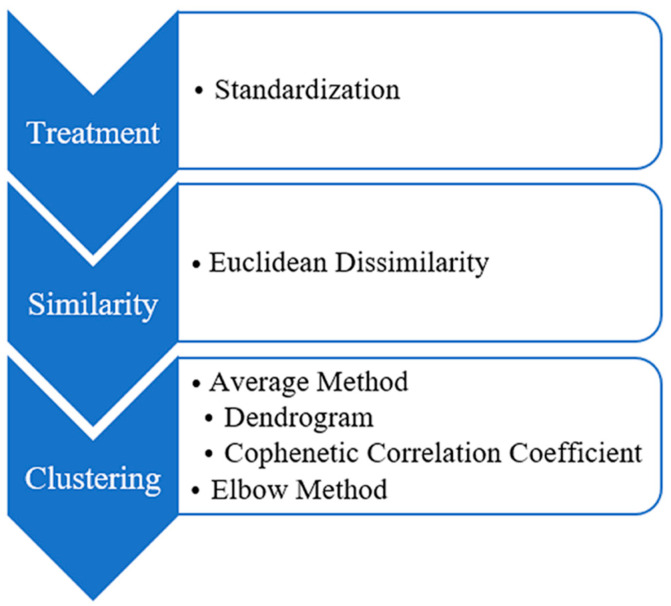
Clustering analysis stages.

In the multivalued analysis we used a dataset without labels or any kind of information about how the instances should be manipulated; we chose unsupervised machine learning, with the cluster analysis method. The Clustering analysis stages adopted are illustrated in Figure 1. The first step was used in all data, before any analysis was performed, to generate the treated and standardized data.

The standardization step was performed when the variables were measured in different units from each other, which may have changed the grouping structure [38]. The z-score technique (Equation (1)) was used, allowing the data to present similar weights for the next step. Each attribute was standardized according to Equation (1),

(1) $$zX=\frac{X-\mu }{\sigma },$$

where *X* represents the original value of the variable, *µ* is the mean of the values associated with the variable, *σ* the standard deviation of the variable, and *zX* the standardized value.

With the standardized data, we defined the similarity coefficient, which can be a measure of similarity or dissimilarity, between the populations to be grouped.

For Fávero et al. [39], measures enable the relationship between the elements, making it possible to observe whether an element A is more similar to B or C. From the measure used, similar elements are grouped, and the others are arranged in distinct groups. We chose the dissimilarity measure, where the greater the value to be observed, the less similar the objects will be, and the distance to be calculated was the Euclidean. According to Manly and Alberto [40] and Han et al. [41], Euclidean distance is the most used measure of distance for cluster analysis. The Euclidean distance between the two patterns, *P_i_* and *P_j_*, in an *m*-dimensional space is defined by Equation (2):

(2) $$d\left(P_{i},P_{j}\right)=\sqrt{\sum_{k=1}^{m}{\left(P_{i_{k}}-P_{j_{k}}\right)}^{2}},$$

where *d*(*P_i_,P_j_*) is the distance between elements *P_i_* and *P_j_*; $P_{i_{k}}\;$ is the value of the indicator *P_i_*, for element *P_i_*; $P_{j_{k}}$ is the element value for the observation *P_j_*; the sum is performed for all indicators (*p*) considered.

The next step was the choice of the grouping or agglomeration process. There are numerous methods of grouping. The differences between the methods exist due to different ways of defining proximity between an individual in a group containing several individuals, or between groups of individuals. Some methods were tested, and the hierarchical average method was chosen; this calculates the average distance between pairs of patterns belonging to different groups. This method was chosen because it presented better results in all executions.

For Everitt and Dunn [42], this method presents good results, and the dissimilarities between the groups are statistically consistent. Among the tests performed, this method showed the best cophenetic results. Equation (3) presents the formula of the average method where |*C_i_*| and |*C_j_*| are, respectively, the number of objects of the groups *C_i_* and *C_j_*, and *x_a_* and *x_b_* are, respectively, the patterns of classes *C_i_* and *C_j_*. Equation (3):

(3) $$d\left(C_{i},C_{j}\right)=\frac{1}{\left|C_{i}\right|\left|C_{j}\right|}\;\underset{\begin{array}{c} x_{a}\in \;C_{i}\\ x_{b}\in \;C_{j}\; \end{array}}{\sum }d\left(x_{a},x_{b}\right).$$

This technique has, as a particularity, the graphical representation in tree format that presents the hierarchy of the obtained partitions called a Dendrogram or Tree Diagram [41]. The dendrograms were generated as a way of visualizing the groups of Brazilian states, and later the map of Brazil of these groups was created. It is possible to assess the degree of deformation caused by the construction of the dendrogram by calculating the cophenetic correlation coefficient (*CCC*) [43,44], which measures the degree of adjustment between the elaborated dendrogram and the dissimilarity matrix. Therefore, we chose to use the *CCC* in this study (Equation (4)):

(4) $$CCC=\frac{Côv\left(F,C\right)}{\sqrt{V\left(F\right)V\left(C\right)}},$$

where *F* represents the phenetic matrix and *C* depicts the cophenetic matrix.

The lower degree of distortion of the dendrogram will be reflected by the higher cophenetic coefficient, provided by the phenetic matrix *F*, in which its values were obtained from the initial distance matrix and by the cophenetic matrix *C*, these being the values obtained from the final distance matrix. The largest *CCC* can better demonstrate the structure of the data, that is, the existence of groups. According to Sokal and Rohlf [44], a *CCC* greater than 0.7 indicates a good adjustment of the grouping method.

The Elbow method, from the non-hierarchical K-means clustering algorithm [45], was also used, to corroborate the results of the dendrograms. This method is used when the ideal number of groups is not known a priori. The value of *k* (number of clusters) is incremented until the sum of the squared distances of the samples against the respective clusters begins to vary very slowly. To calculate this method, the Sum of Squared Error (*SSE*) is used, according to Syakur et al. [45] (Equation (5)):

(5) $$SSE=\sum_{k=1}^{k}\sum_{X_{j}\in S_{k}}{\left|X_{j}-C_{k}\right|}^{2},$$

where *k* is the number of originated groups; *C_k_* is the *i*-th cluster; *X* refers to the data in each group.

Data were processed using Python programming language, and Tableau (version 2021.3.3, Tableau Software) was used for data analysis and visualization. For each PQ, cluster analysis was performed, to later carry out the discussions.

## 3. Results

### 3.1. Trends Analysis of Infodemic Term Searches

**Figure 2:**
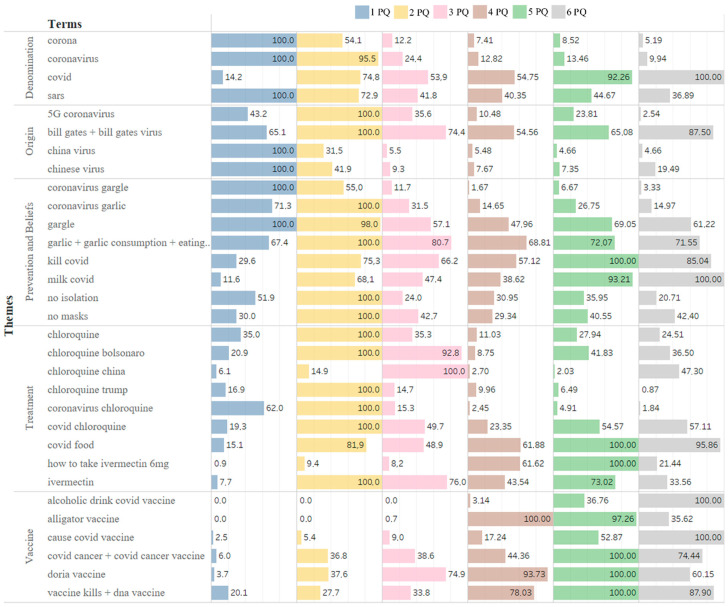
Search trend for infodemic terms in Brazil in RSVs.

The search response performed in GT shows how often a particular search term is entered into the Google search engine in relation to the total search volume in a given period. In this sense, we can see concrete changes in Brazil in all categories (Figure 2), some searches maintaining a pattern throughout the quarters and others more punctual as the pandemic evolved and new information about it was introduced in the world and Brazilian society.

The “Denomination” theme is based on the nomenclature used for the SARS-CoV-2 virus that causes the COVID-19 disease, with the term “COVID” predominating in searches since April 2020, a simpler way of writing and searching. The other terms had considered searches but in a smaller volume. We consulted searches in an earlier period, 2017 to 2019, and there were no searches for the term “coronavirus” or “corona” (which were already known terms for over 20 years), and only sixty-nine RSV searches for the term “SARS”.

The terms involving the “Origin” of the virus were highlighted in the search for linking the virus with businessman Bill Gates, as he mentioned in an interview (in 2015) the emergence of a pandemic [46]. The term “5G coronavirus” was not searched many times, unlike in other countries, such as the UK, where there was a substantial increase in the hashtag #5GCoronavirus in a social network [19]. However, in Brazil, they also did not have antennas that provided the 5G signal. The terms mentioning “Chinese virus” or “China virus” were not searched for in Brazil either, as shown in other works by Budhwani and Sun [47] and Hu et al. [48].

The next two themes had large volumes of research, which were “Prevention and Beliefs” and “Treatment”. Prevention measures, such as wearing masks and isolation, had national prominence when the President of Brazil, Bolsonaro, did not respect these protocols indicated by the WHO and in some pronouncements, he stated that “The effectiveness of this mask is almost zero” [10]. Also, searches for how to prevent the virus through the consumption of some foods and gargling were constant in the quarters. The highlighted search was “garlic + garlic consumption + eating raw garlic is bad”, which was already a constant search in Brazil; during the pandemic, however, many searches brought results relating to food, such as prevention and treatment of COVID-19.

In Brazil, the use of drugs such as chloroquine and ivermectin were indicated by some state authorities and the President of Brazil as early treatments for COVID-19, even without scientific studies. The president, in an interview in October 2021, said that if he became infected again, he would undergo treatment using chloroquine and ivermectin [49]. This search increased over the months, as well as foods to treat the disease.

In the Vaccine category, searches began to be highlighted as of April 2020 when the governor of São Paulo, João Doria, had already spoken of a vaccine (“Doria vaccine”) in many statements, being in its favor. President Bolsonaro, in addition to making fun of the vaccines with comments that “they don’t work”, or “the vaccine is not Doria’s”, said (in December 2020) that the vaccine could turn the person into an alligator [31]. Consequently, the ‘alligator vaccine’ was highly researched from that date. Subsequently, infodemic information such as “vaccination kills”, “vaccine causes COVID” increased in the year 2021, when the vaccination campaign was already starting in Brazil, the first vaccine being given on 17 January 2021.

### 3.2. Exploratory Spatial Analysis of Brazilian States

We used an exploratory spatial analysis, visualizing the data on geographic maps of Brazil, allowing for a better understanding of the spatial dynamics existing in the data studied, in the period between January 2020 and June 2021.

#### 3.2.1. First Scenario—Infodemic and Variables That Illustrate Social Inequality

**Figure 3:**
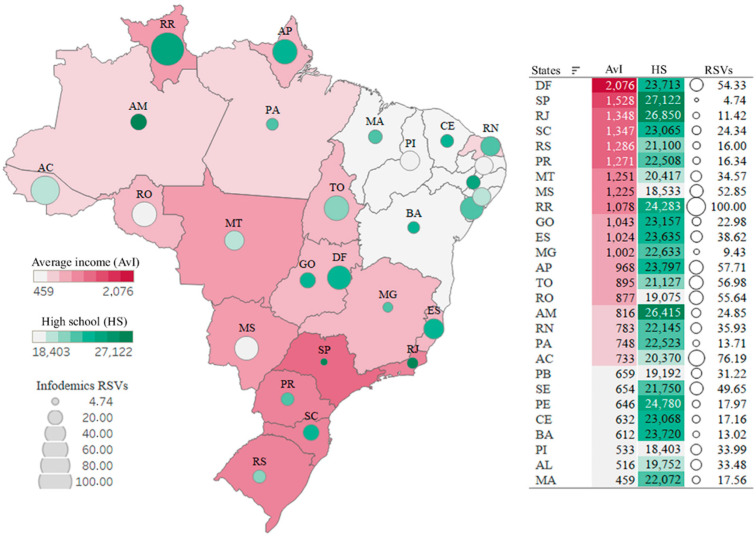
Spatial distribution: Infodemics RSVs, income, and education.

A map of Brazil is shown in Figure 3, with data (RSVs) from the infodemic in the COVID-19 pandemic, as well as socioeconomic variables, such as average per capita income and high school education level, referring to the social reality of each state. Brazil has a share of 31.8% (around 65 million) of its population living in poverty or extreme poverty, and 27. 4% of the population with a high school education [50,51].

Looking at the map, it is possible to see a very large income inequality (represented in red), ranging between R$2076 and R$459, the highest income in the Federal District (DF), and the lowest in the state of Maranhão (MA). We used a circle-size scale for the intensity of searches for infodemic terms, and the color green, of different intensities (lighter to darker), to represent the level of education as having completed high school.

Note that high-income states and low-income states have similar numbers, and low-income states are equal in the number of infodemic RSVs. Comparing the two extreme states of income data, the search for the infodemic in the DF is more than double, compared with that in the MA. States with low or high rates of high school completion had similar numbers of searches for infodemic information. It is possible to visualize and perceive information from similar infodemic RSVs, referring to the data for each state, even those states showing many variations between income and education, as in DF and SE, and PA and MG.

#### 3.2.2. Second Scenario—Infodemic and Impact of Social Programs

**Figure 4:**
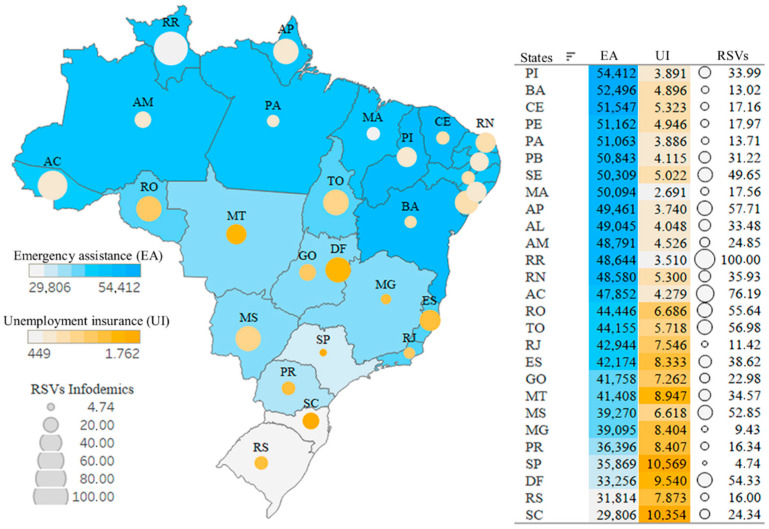
Spatial distribution: Infodemic RSVs and Government Programs.

For an analysis of government income programs used in the pandemic, we plotted on the map in Figure 4 RSVs of infodemic terms on the rates of emergency assistance payments (starting in April 2020) and unemployment insurance claims, which in the first quarter of 2020 had an increase of almost 20% compared with the last quarter of 2019 and an increase of almost 125% from 2 PQ to 1 PQ.

Far from being homogeneous, Brazilian states portray the inequality in requests for government programs, where states in the north and northeast asked for more emergency assistance, and states in the south, southeast and midwest have more unemployment insurance, while queries for infodemic terms were observed in similar numbers in states with widely dispersed government program indexes.

**Figure 5:**
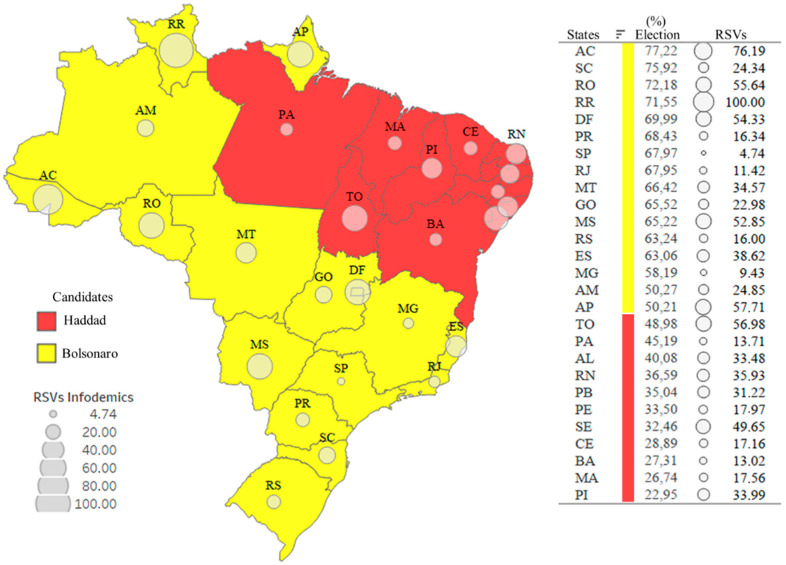
Spatial distribution: Infodemic RSVs and 2018 presidential elections.

It is understood that the tendency of the Brazilian population to search for false information is not related to any remarkable or particular characteristic, such as a state with high or low income, high or low education, or government programs (as can be seen in Figure 4 and Figure 5).

#### 3.2.3. Third Scenario—Infodemic and Public Influences

To analyze one of the issues raised in the work, the spatial dynamics of data from the 2018 presidential elections in Brazil was carried out. In the second round, two candidates, Jair Bolsonaro and Fernando Haddad, were running. Jair Bolsonaro was elected. The importance of a leader and his attitudes and actions can influence an entire nation. Leaders can affect individuals’ beliefs and behavior through different channels [52].

In this context, Figure 5 shows the states in yellow (a total of sixteen), which are the states in which Jair Bolsonaro obtained the highest number of votes (>50%), and where there are more searches for infodemic terms. There are 46.58% more searches in these states. The states with the most Infodemic RSVs are in the top four by percentage vote for the President. Analyzing Figure 4 and Figure 5, the states where Bolsonaro received the least number of votes (<50%) were the states where there was a higher payment of emergency assistance.

### 3.3. Cluster-Based Multivalued Analysis

To understand the patterns of infodemic research by the Brazilian population in some scenarios, we chose to use a multivalued analysis technique, the Cluster Analysis, having a visualization of similar patterns in different socio-demographic regions of Brazil.

In the first two scenarios, the technique was applied in each PQ, generating the grouping of similar patterns in the variables presented in each situation. The third scenario was analyzed considering the infodemic terms researched throughout this study (January 2020 to June 2021). All the generated dendrograms are in the Supplementary Material S2 and the images of the map of Brazil were used to better visualize the results of the clustering techniques.

#### 3.3.1. First Scenario—Infodemic and Variables That Illustrate Social Inequality

For the analysis of this scenario, regarding events in the evolution of the pandemic and attitudes of the population, we cite some highlighted variables of social inequality, such as average quarterly per capita income and level of education as having completed high school, as well as internet access, research trends in the population for infodemic terms, and COVID-19 numbers (deaths and case numbers).

**Figure 6:**
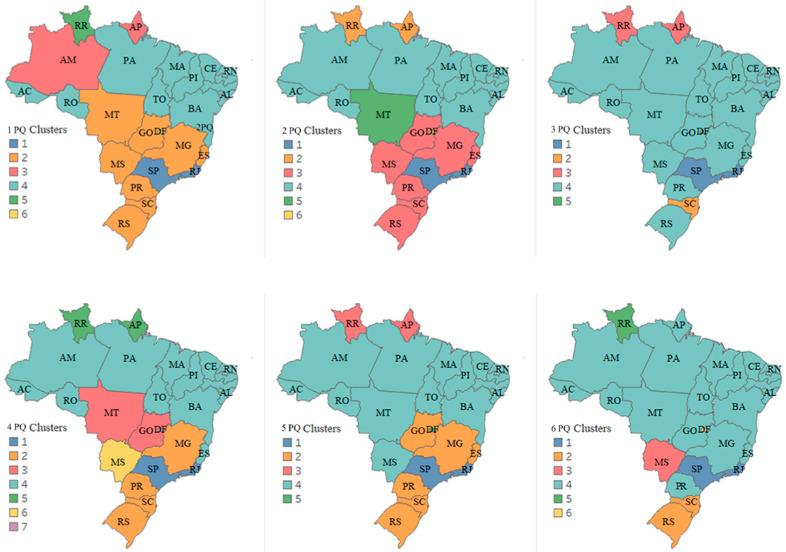
Clustering of Brazil in the pandemic quarters—Infodemic and social inequality. States assigned to the same cluster share the same color.

The first three maps in Figure 6 show the 1 PQ, 2 PQ, and 3 PQ, from January to September 2020, encompassing the first wave of the pandemic in Brazil. Based on the dendrograms generated, it is observed that in the 1 PQ and 2 PQ, six groups are considered, and in the 3 PQ, there are five groups, with different numbers of states in each group. The most similar states, based on the analysis, are São Paulo and Rio de Janeiro, Amapá and Roraima, and the Federal District, which, in every dendrogram, formed a single group. It is assumed that these groups have greater similarities to each other in terms of behavior for the observed period, and distinct characteristics from the others.

The *CCC* of the generated dendrograms are 0.89, 0.88, and 0.91, respectively, indicating satisfactory adjustments between the graphical representation (dendrogram) and the dissimilarity matrix, making it possible to perform inferences through the visual analysis of the dendrogram. The Elbow method, also used in the technique, provides the number of clusters in 6, 6, and 5 for the 1 PQ, 2 PQ, and 3 PQ quarters.

In Brazil, the disease affected the regions differently, which can be seen on the map, where in the 1 PQ we have six clusters, with States 2, 8, 2, 13, 1, and 1, respectively, and we noticed that over the months the patterns change. The onset of the pandemic (some reported cases) took place, first in São Paulo, then in Rio de Janeiro, and after two weeks, some states in the southeastern, northeastern, midwestern, and southern regions presented cases of COVID-19. In March, the first cases were reported in Amazonas: this northern region would become an epicenter of the pandemic from April 2020. The 2 PQ clustering shows that the patterns started to become more similar, as the population received a tsunami of information, not all from reliable sources, and the population needed to search, read and understand what was going on with this new and deadly disease.

In mid-June, the northeastern region had an increase in the number of cases. Only from July 2020, 3 PQ, the midwestern region, which at the beginning of the pandemic was little affected by the outbreak, had a significant increase in the number of cases, followed by the southern region [53]. The 3 PQ contains five clusters and one of them has the highest number of clustered states, twenty-one in all. Based on Figure 6, the population’s tendency to look for infodemic information is independent of the income of each state and the level of education but seems related to noticing the growth of the pandemic, and significant increases in the number of cases and deaths.

In the groupings of the following quarters in Figure 6 (4 PQ, 5 PQ, and 6 PQ), the most similar states, based on the analysis, are São Paulo and Rio de Janeiro, Amapá and Roraima, and Federal District which in all dendrograms formed a group alone. The *CCC* of the generated dendrograms are 0.90, 0.87, and 0.91, respectively, indicating a satisfactory fit between the graphical representation (dendrogram) and the dissimilarity matrix.

In mid-September 2020, there was a descending curve in the first wave of the pandemic, which may have influenced the grouped patterns for the 4 PQ, with seven clusters, the highest number so far in the study. Also, in October there were elections in the Brazilian states, modifying the population’s attitudes focused on interests in the elections, such as, for example, crowds of people due to political party meetings and street marches. In December 2020, with the appearance of new variants, mainly the Delta and Gama variants, the latter identified for the first time in Manaus (capital of the Amazon), modified the 5 PQ and 6 PQ scenarios. As of January 2021, there was a significant increase in the number of cases and deaths in all Brazilian regions, presenting a more overwhelming scenario, in the number of deaths, than in 2020, already encompassing the second wave of the pandemic.

False information continued to circulate on social networks and media, with more emphasis on vaccination. According to a survey by the Datafolha Institute [54], the percentage of Brazilians willing to be vaccinated against the disease dropped from 89% in the first half of August to 73% in December, and the share of people who declared they did not want to have the vaccine increased from 9 to 22%. In the group that always trusts the statements of President Jair Bolsonaro, 61% are against mandatory vaccination of all Brazilians. In the 5 PQ and 6 PQ, the total number of clusters is five and six, returning to concentrate more states in a single cluster, thus characterizing a similar pattern in many states.

Based on the results, the Federal District is always evidenced as an isolated cluster, Rio de Janeiro and São Paulo forming a single cluster, and an emphasis on the State of Roraima, which was always in a single cluster or shared with Amapá. The state of Roraima presented results alone or with another state. Analyzing this state, it has remarkable characteristics, with median income, and high rates in the other variables studied, maintaining a constant in the results.

#### 3.3.2. Second Scenario—Infodemic and Impact of Social Programs

**Figure 7:**
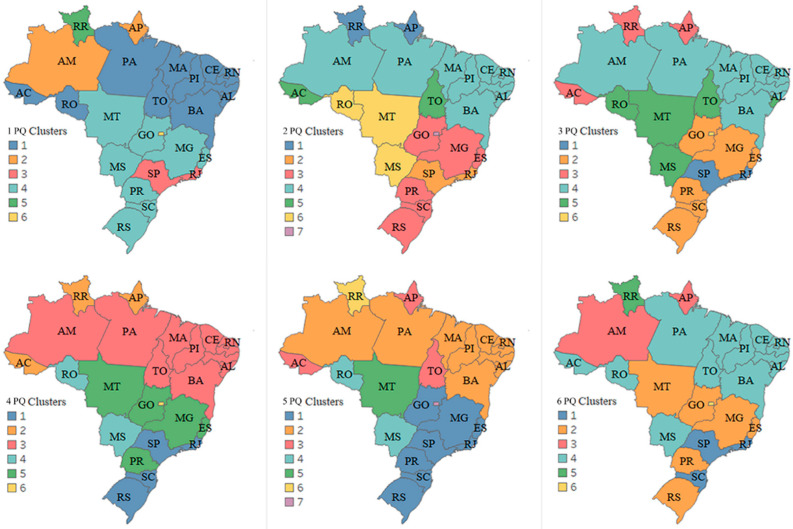
Clustering of Brazil in the pandemic quarters—Infodemic and government cash transfer programs. States assigned to the same cluster share the same color.

In this scenario, the intention is to analyze the clusters generated by PQ with the impact of government income transfer programs. Two variables were added to the analysis, unemployment insurance numbers, and emergency assistance numbers. The generated maps are shown in Figure 7.

The multivalued analysis technique was applied in each PQ, generating clusters in the maps from the dendrograms and with satisfactory *CCC* (0.82, 0.78, 0.78, 0.82, 0.80, and 0.77, respectively, for each PQ) and clustering Brazil in a different format from the previous scenario, despite presenting a similar number of clusters with 6, 7, 6, 6, 7 and 6, respectively, for each PQ. The maps indicate research groups with similar characteristics when there is a need to understand and obtain the programs that make up public employment and income transfer policies to protect workers who live in informality while activities are at a standstill [55].

It was observed that from the 2 PQ to the 5 PQ northern and northeastern states have similar search patterns, as does the southern region. According to Barros [56], the effects of the COVID-19 pandemic on the labor market record high unemployment rates in Brazil, with the highest unemployment rates registered in the northeastern states and the lowest in the south of the country. The IBGE survey indicates that the average unemployment rate in 2020 was a record in twenty states of the country, following the national average, which increased from 11.9% in 2019 to 13.5% last year, the highest in the historical series of the Continuous PNAD, which started in 2012 [56]. Self-employment also broke a record in the country, where out of every ten new jobs created in the country in the last year, seven were self-employed [57], which can influence the numbers of clusters generated in 2020 and 2021.

The Federal District once again presented particular characteristics, as did the states of Roraima and Amapá, Rio de Janeiro, and São Paulo. These last two that formed a single group, formed groups with other states from 2021. In the 6 PQ, it can be seen that the number of states per cluster decreases, which coincides with an improvement in the unemployment rate, with one of the lowest rates since the beginning of the pandemic, at 14.1% [58].

#### 3.3.3. Third Scenario—Infodemic and Public Influences

In Brazil, despite all the work of scientists and health professionals, many political authorities denied prevention measures and published an infodemic. In this scenario, this third clipping sought to identify the impact of the President of the Republic’s influence in searches for infodemic terms. We used data from the 2018 elections and RSVs from infodemics during the pandemic, until 30 June 2021.

**Figure 8:**
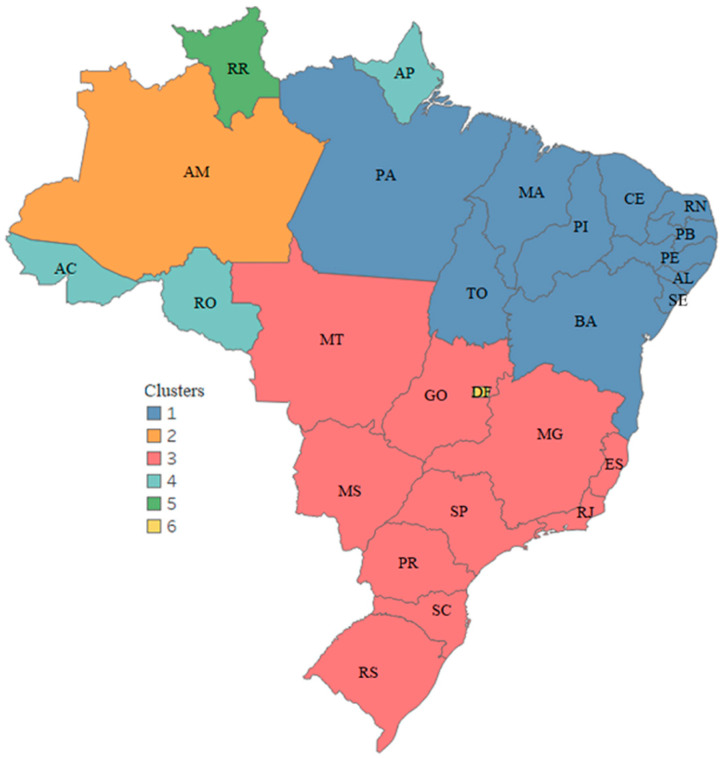
Clustering of Brazil—Infodemic × president of the republic. States assigned to the same cluster share the same color.

The multivalued analysis technique was applied to national data, generating six clusters on the map (Figure 8) from the executed dendrogram and with *CCC* = 0.86, and clustering Brazil in a similar behavior format. Cluster 1 refers to the states where the President was not the most voted-for in 2018, presenting indicators with more similar patterns in searches for infodemic terms and when presented in quantitative numbers, less influenced by the Political Leader.

The state of Amazonas (AM) and Federal District (DF) formed a separate group, being the states with the lowest and highest density of internet accesses, respectively. Cluster 3 has ten states, and comprises three Brazilian regions, southern, southeastern, and midwestern, concentrating the highest incomes in the country. The other clusters present groups of states with similar behavior in searches for infodemic terms.

## 4. Discussion

Among the most researched infodemic themes, those in the Prevention and Beliefs and Treatment categories prevailed. This was because many Brazilians believed in ineffective and scientifically unproven treatments and did not give due importance to the COVID-19 virus and the effectiveness of vaccines, even being encouraged to do so by political authorities. India, for example, which ranks second in the number of cases and third in the number of deaths, announced on 24 September 2021, through the Indian Council of Medical Research, the removal of chloroquine and ivermectin from its list of approved drugs for the treatment of COVID-19. On the other hand, Brazil is third in the number of cases, and second in the number of deaths; nevertheless, the Brazilian President said on 14 October that if he was re-infected by SARS-CoV-2, he would follow the so-called early treatment using chloroquine and ivermectin.

The analysis shows that research on infodemic information consistently stands out, regardless of Brazilian region. Socioeconomic variables, such as the population’s monthly income or the same level of education, were used to identify possible impacts on research patterns and it was observed through the results of dendrograms, visualized on the maps, that states with a high or low monthly income have similar patterns, staying together in the same clusters.

Government income programs were also evaluated, and it was noticed that in parallel with the searches for the programs, infodemic searches about the disease and pandemic continued, since the failures in dealing with the pandemic were not reviewed or corrected by the public authorities. According to the maps, the techniques made it possible to group the northern and northeastern states in almost all quarters, regions where unemployment showed the highest rates. The economic impact due to the COVID-19 pandemic was and still is very large across several sectors.

In addition to the constant infodemic polls in Brazil, the results imply that states where the President was the most voted-for, had more infodemic RSV polls: indeed, 46.58% more where he was not the most voted-for. The president did not set an example of social distancing measures, denied the importance of wearing masks, promoted ineffective early treatment (with chloroquine, ivermectin, and other treatments), and repeated statements about the effectiveness of the vaccine.

The present study has some limitations. By presenting analyses of a period of 18 months and data from different sources, there was a very thorough preparation of the database, with the final base being generated by quarterly data. A better-treated base could reflect more coherent and significant results. Another limitation of this study is that we analyzed GT infodemic terms, which capture the search behavior of people using the Google search engine. Consequently, we did not investigate infodemics discussed on social media. Finally, the use of the presented methodology was tested and revised until obtaining the best coefficients and results.

## 5. Conclusions

Denial, misinformation concerning preventive measures for the disease, indication of early medication, disbelief in science, disqualification of vaccination, the various changes in the management of the Ministry of Health, in addition to the lack of integrated national actions, end by impacting, to a greater degree, the effects of the pandemic, directly reflecting the growing negative results regarding COVID-19 in Brazil and constant searches for infodemic information, as observed in the study and the results of the technique used.

Cluster analysis was essential to create groups with similar patterns to each other and divergent in relation to the other groups, and to understand how the population reacts to events resulting from the pandemic and failures in dealing with it by public authorities, and an excessive infodemic. Thus, it is possible to be more assertive in the formulation of public policies to combat infodemics, which was already seen as a global risk, presented at the 2013 World Economic Forum [59].

The use of GT in health and medicine, focusing mainly on surveillance and analysis of health topics and disease prediction, correlated with target variables for research, such as income and the influence of authorities; these, among others, can generate satisfactory results and contribute to redefining public policies, decreasing the population’s fear of the pandemic, and increasing confidence in the information read, received, and shared.

Future work should combine more information, encompassing more searches for infodemic terms, particularly on the vaccine, and for the entire year of 2021, thus ending two years of the pandemic. Adding other variables that express social and sociodemographic (age, sex) inequality, and in health areas, such as vaccination numbers, would provide more scenarios of the behavior of the Brazilian population, which would help policymakers and health managers to plan health resources and control the prevention of outbreaks, and prevent the spread of an infodemic.

## Figures and Tables

**Table 1:** Pandemic quarters.

Year	Period	Pandemic Quarters
2020	January to March	1 PQ
	April to June	2 PQ
	July to September	3 PQ
	Octorber to December	4 PQ
2021	January to March	5 PQ
	April to June	6 PQ

## Data Availability

Data are available at github, accession link: https://github.com/mpenhaharb/Analysis-GT (accessed on 14 January 2022).

## Supplementary Materials

The following supporting information can be downloaded at: https://www.mdpi.com/article/10.3390/ijerph19063208/s1, Supplementary Materials S1: Information on Brazilian states and their regional divisions; Figure S1: Brazilian states; Figure S2: Geographic regions; Supplementary Materials S2: All the generated dendrograms; Figure S3: Dendrogram—1 PQ—First Scenario; Figure S4: Dendrogram—2 PQ—First Scenario; Figure S5: Dendrogram—3 PQ—First Scenario; Figure S6: Dendrogram—4 PQ—First Scenario; Figure S7: Dendrogram—5 PQ—First Scenario; Figure S8: Dendrogram—6 PQ—First Scenario; Figure S9: Dendrogram—1 PQ—Second Scenario; Figure S10: Dendrogram—2 PQ—Second Scenario; Figure S11: Dendrogram—3 PQ—Second Scenario; Figure S12: Dendrogram—4 PQ—Second Scenario; Figure S13: Dendrogram—5 PQ—Second Scenario; Figure S14: Dendrogram—6 PQ—Second Scenario; Figure S15: Dendrogram—Third Scenario.
