# Supplementary material for: An Analysis of the Deleterious Impact of the Infodemic during the COVID-19 Pandemic in Brazil: A Case Study Considering Possible Correlations with Socioeconomic Aspects of Brazilian Demography
Source: Int J Environ Res Public Health. 2022 Mar 9;19(6):3208. doi: 10.3390/ijerph19063208 (PMC8953409; doi:10.3390/ijerph19063208)
Supplement: Supplementary file 1 [file ijerph-19-03208-s001.zip › Supplementary Materials S1.pdf]

Supplementary Materials S1: Information on Brazilian states and their regional divisions

Brazil is the fifth largest country in the world geographically, and the sixth in population, has 5,570 municipalities divided into 27 federative units (26 states and the Federal District) (Figure S1), which are grouped into five geographic macro-regions (Midwest, Northeast, North, Southeast and South) (Figure S2).

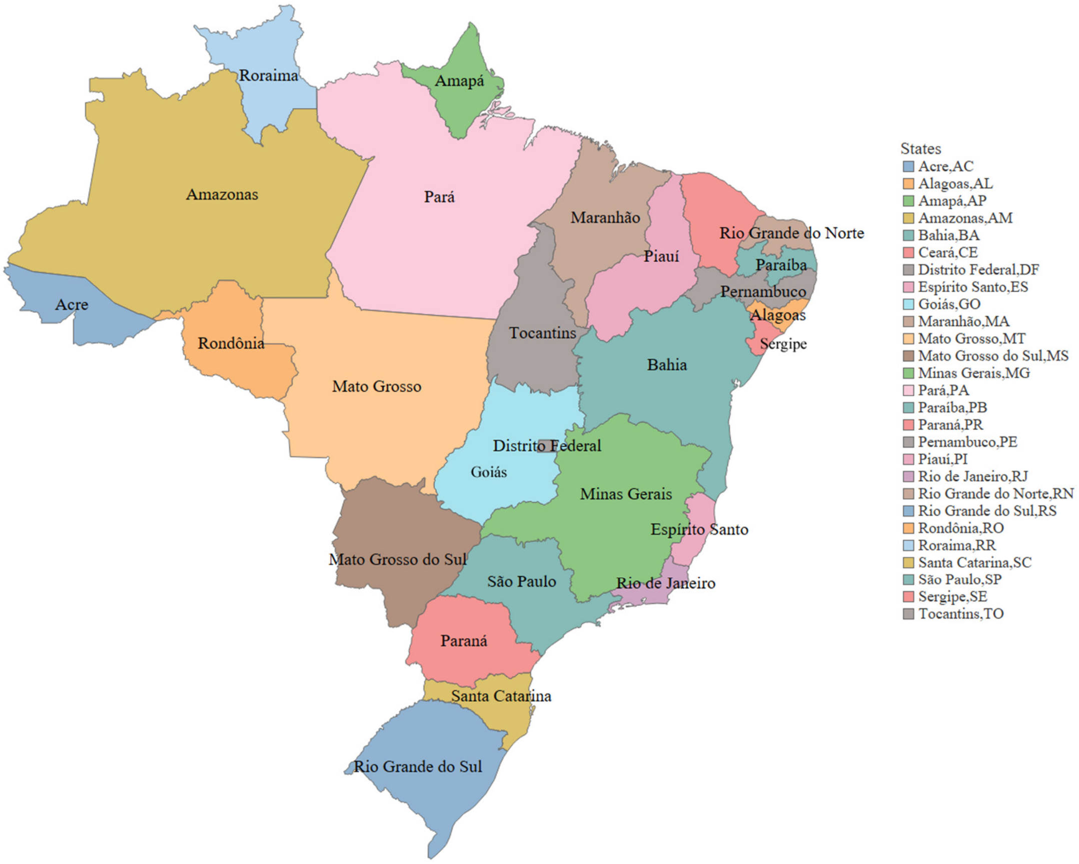

Figure S1. Brazilian states.

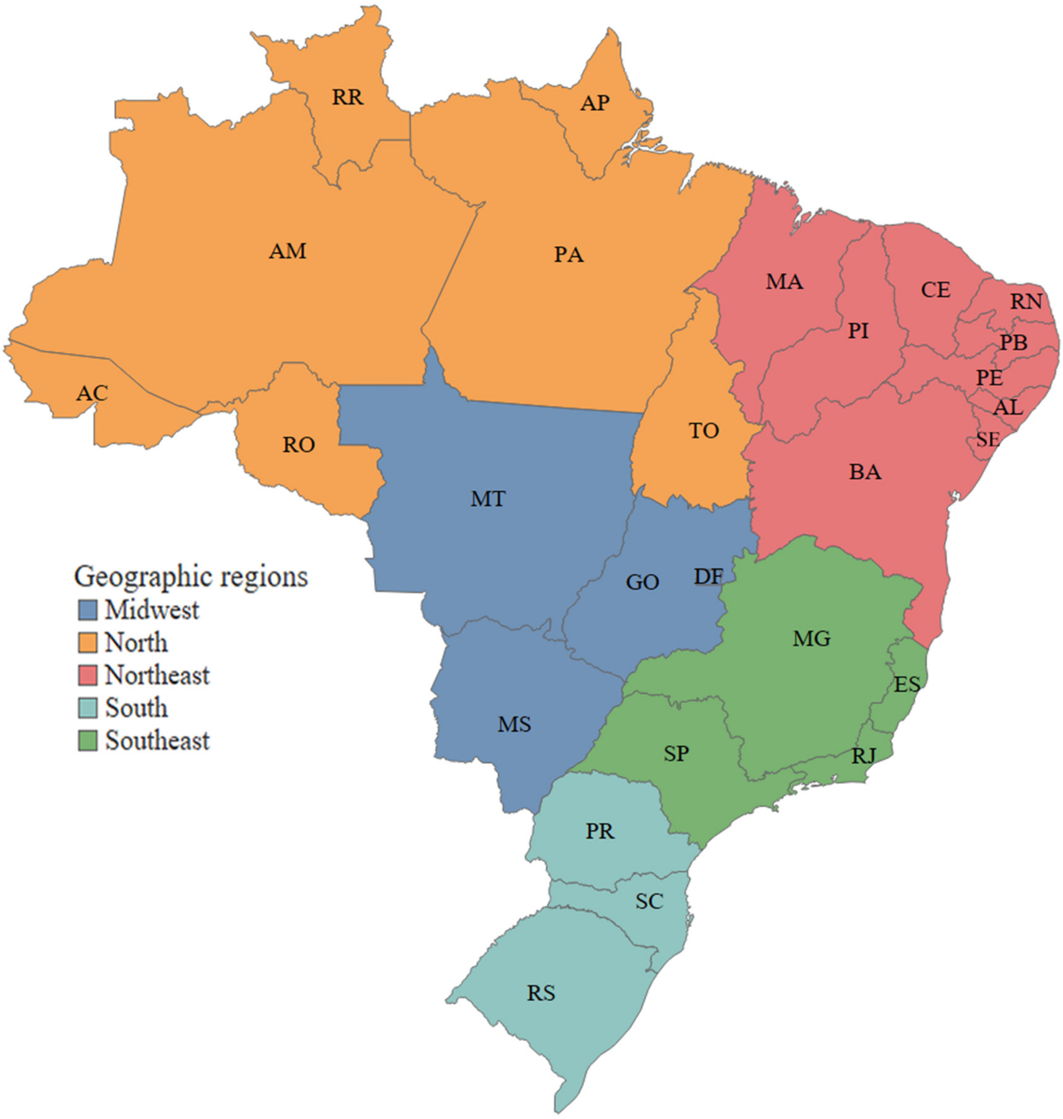

Figure S2. Geographic regions.
