# Supplementary material for: An Analysis of the Deleterious Impact of the Infodemic during the COVID-19 Pandemic in Brazil: A Case Study Considering Possible Correlations with Socioeconomic Aspects of Brazilian Demography
Source: Int J Environ Res Public Health. 2022 Mar 9;19(6):3208. doi: 10.3390/ijerph19063208 (PMC8953409; doi:10.3390/ijerph19063208)
Supplement: Supplementary file 1 [file ijerph-19-03208-s001.zip › Supplementary Materials S2.pdf]

Supplementary Materials S2: Generated dendrograms on cluster-based multivalued analysis

First Scenario – Infodemic and variables that illustrate social inequality

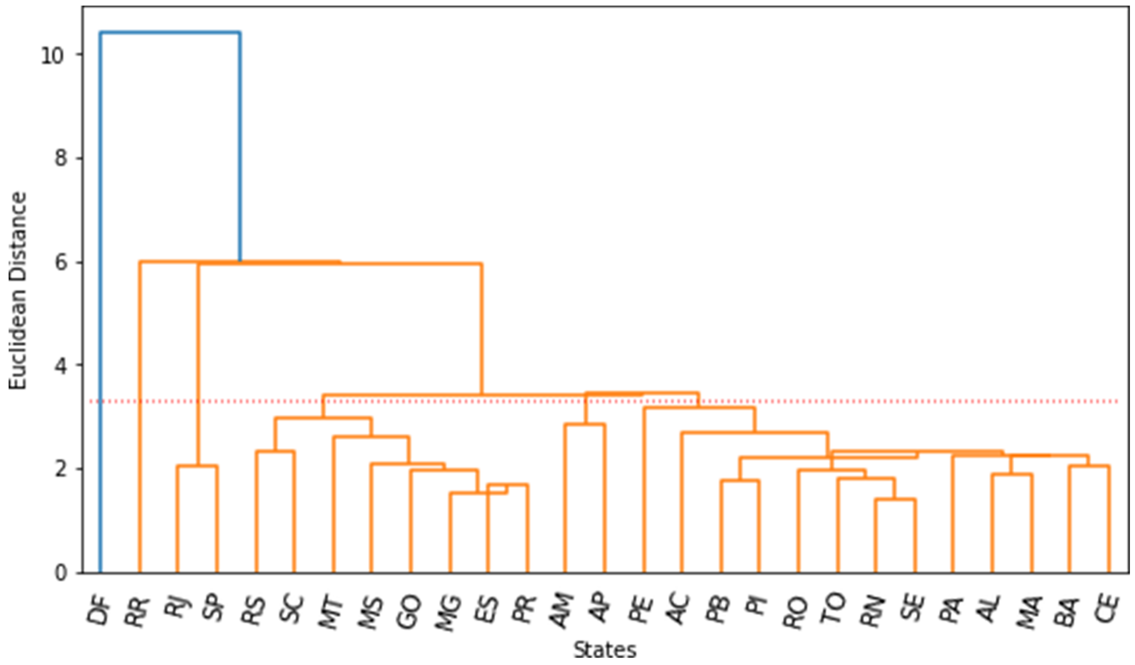

Figure S3. Dendrogram – 1 PQ - First Scenario.

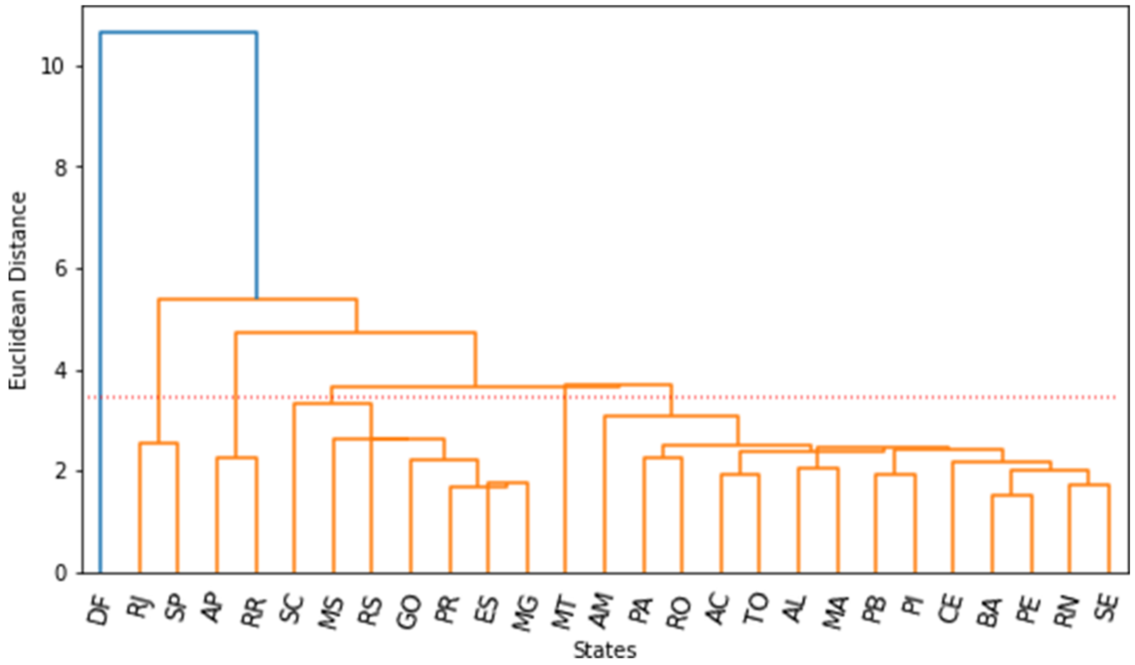

Figure S4. Dendrogram – 2 PQ - First Scenario.

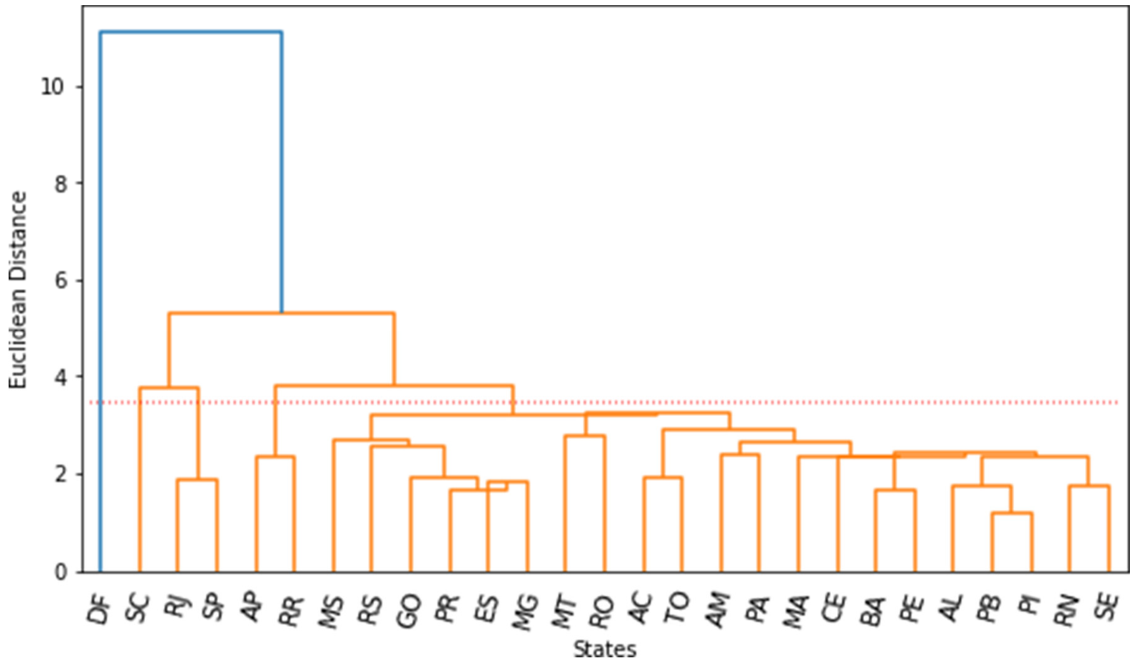

Figure S5. Dendrogram – 3 PQ - First Scenario.

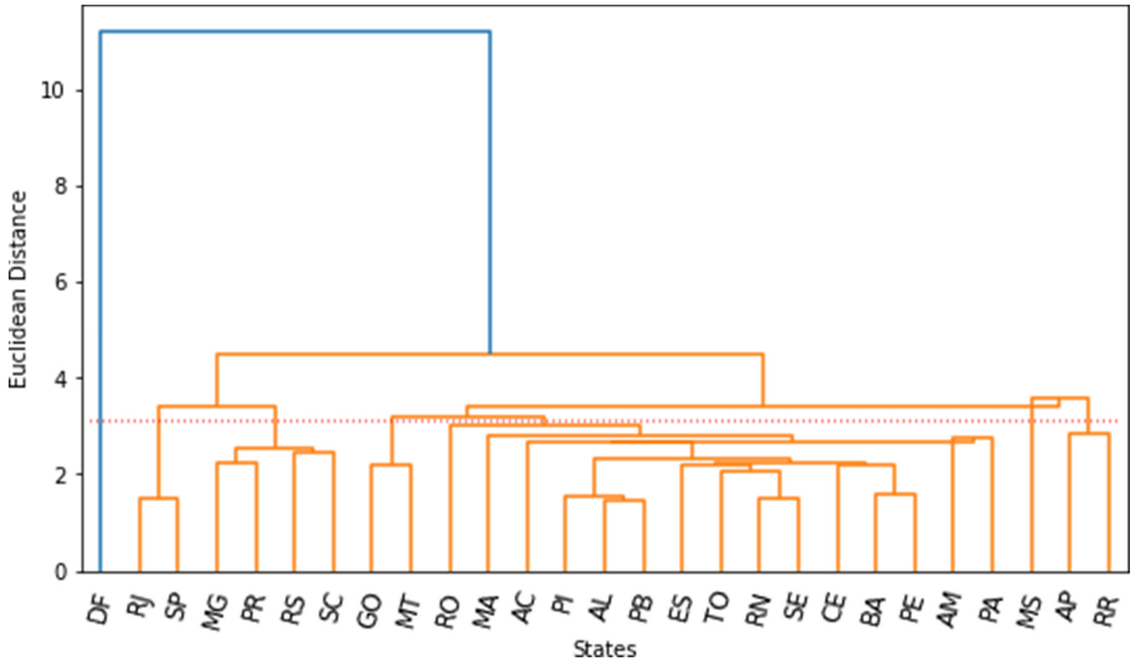

Figure S6. Dendrogram – 4 PQ - First Scenario.

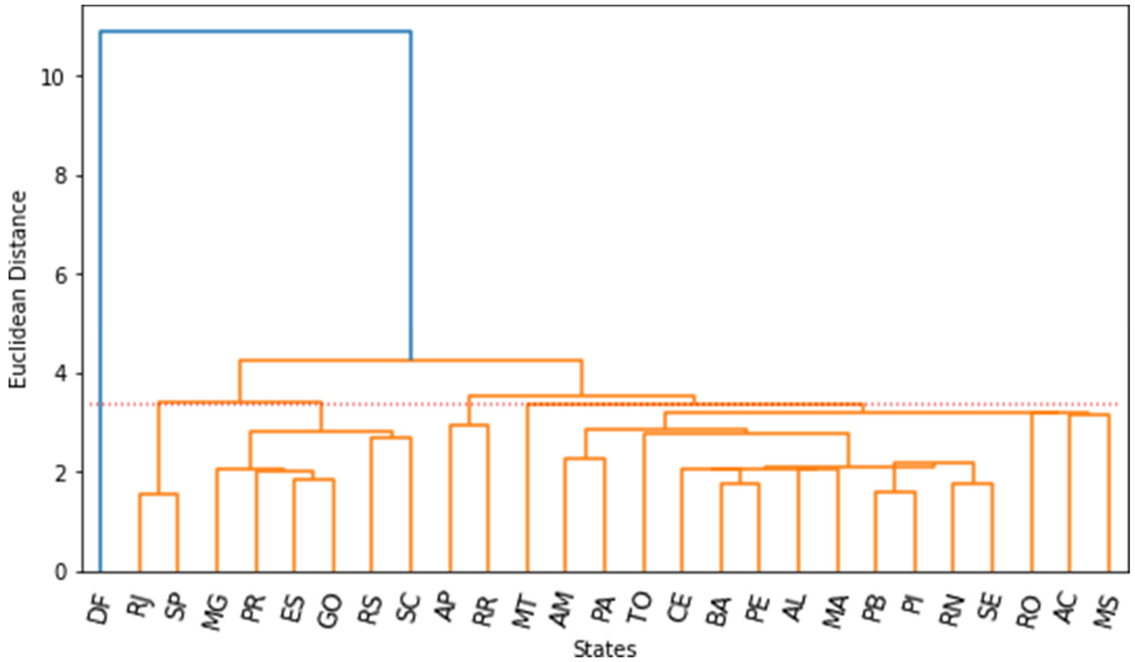

Figure S7. Dendrogram – 5 PQ - First Scenario.

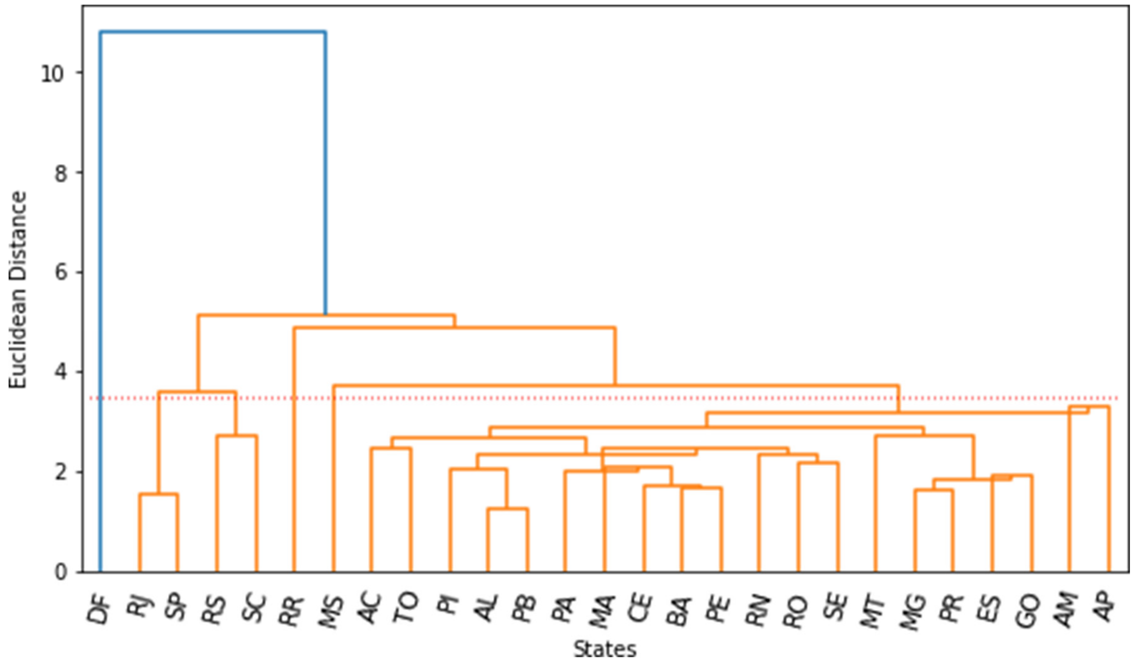

Figure S8. Dendrogram – 6 PQ - First Scenario.

Second Scenario – Infodemic and Impact of social programs

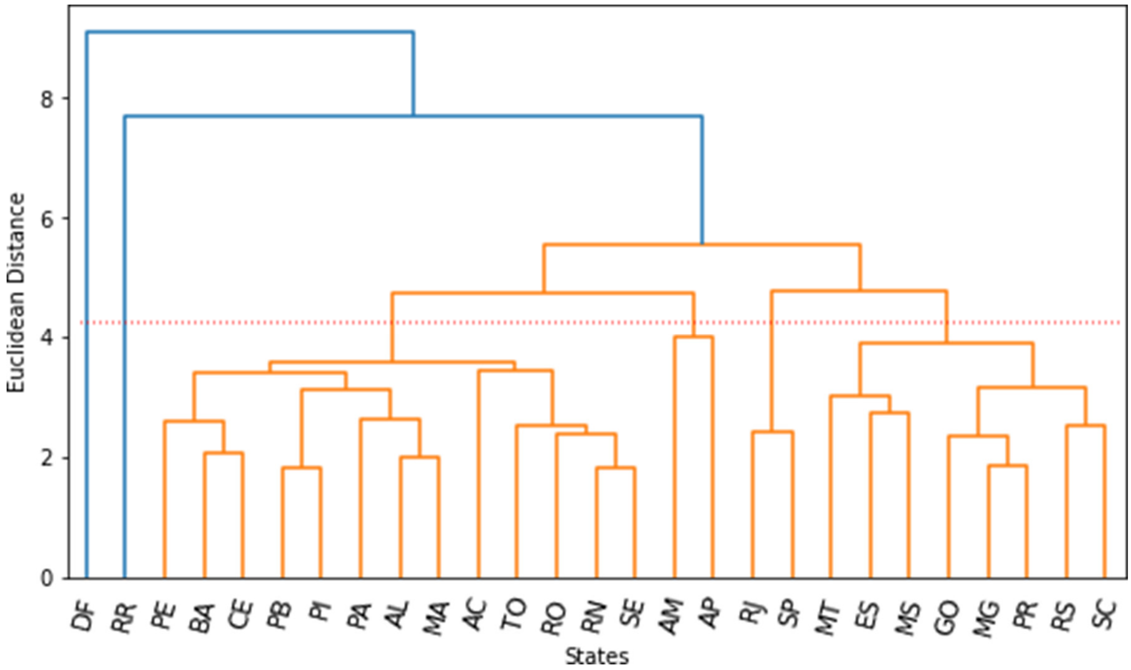

Figure S9. Dendrogram – 1 PQ - Second Scenario.

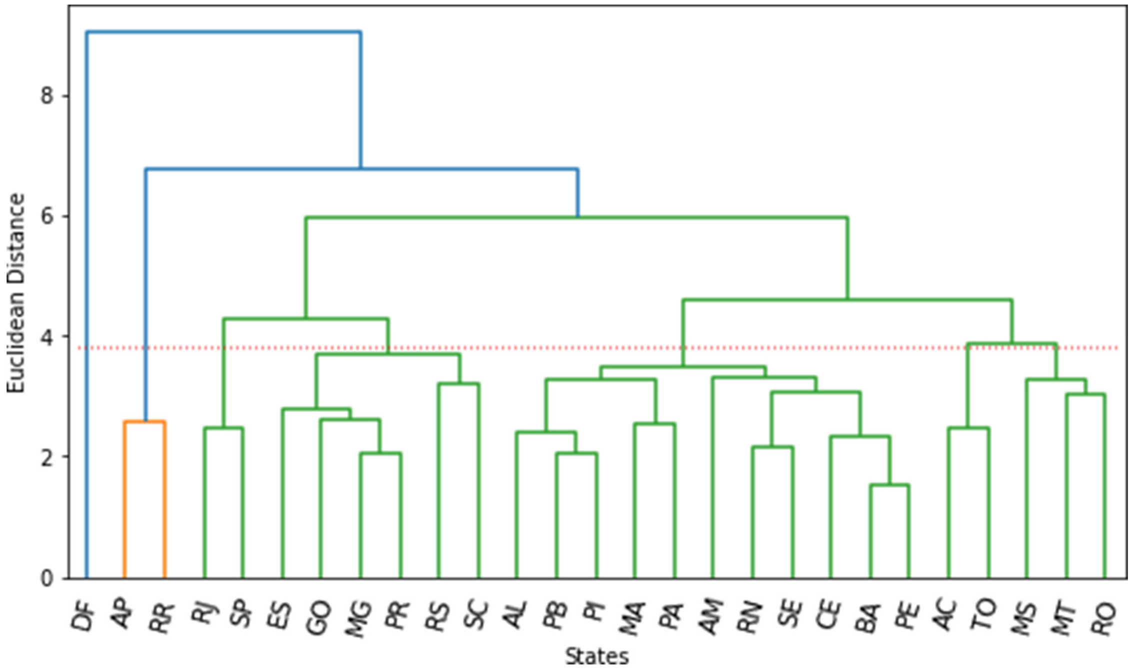

Figure S10. Dendrogram – 2 PQ - Second Scenario.

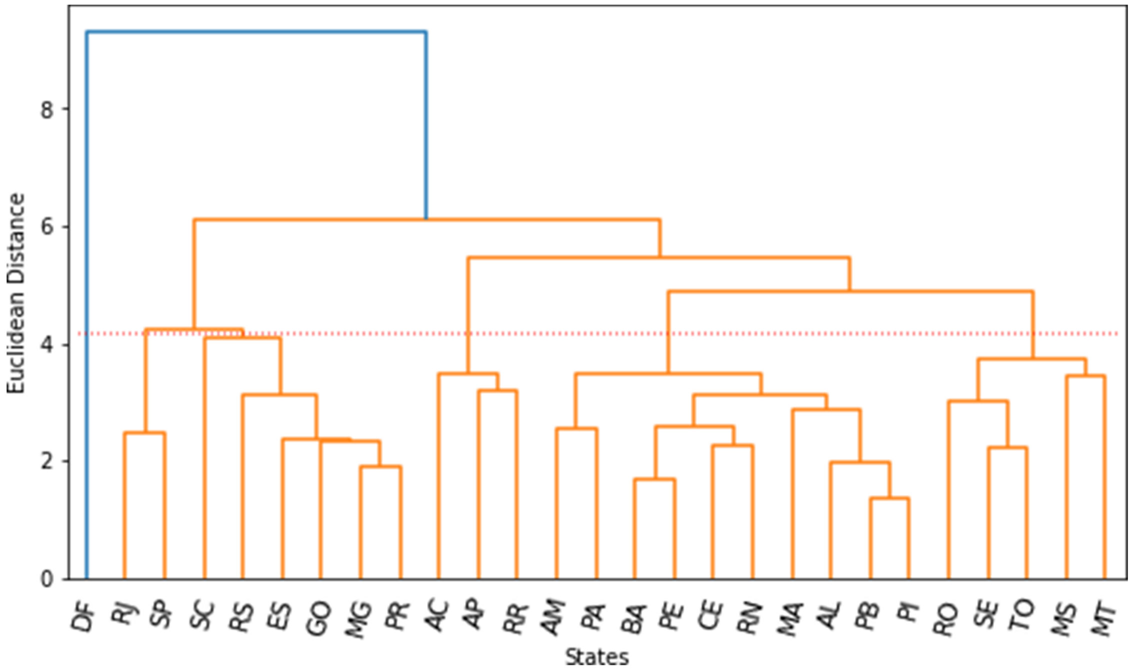

Figure S11. Dendrogram – 3 PQ - Second Scenario.

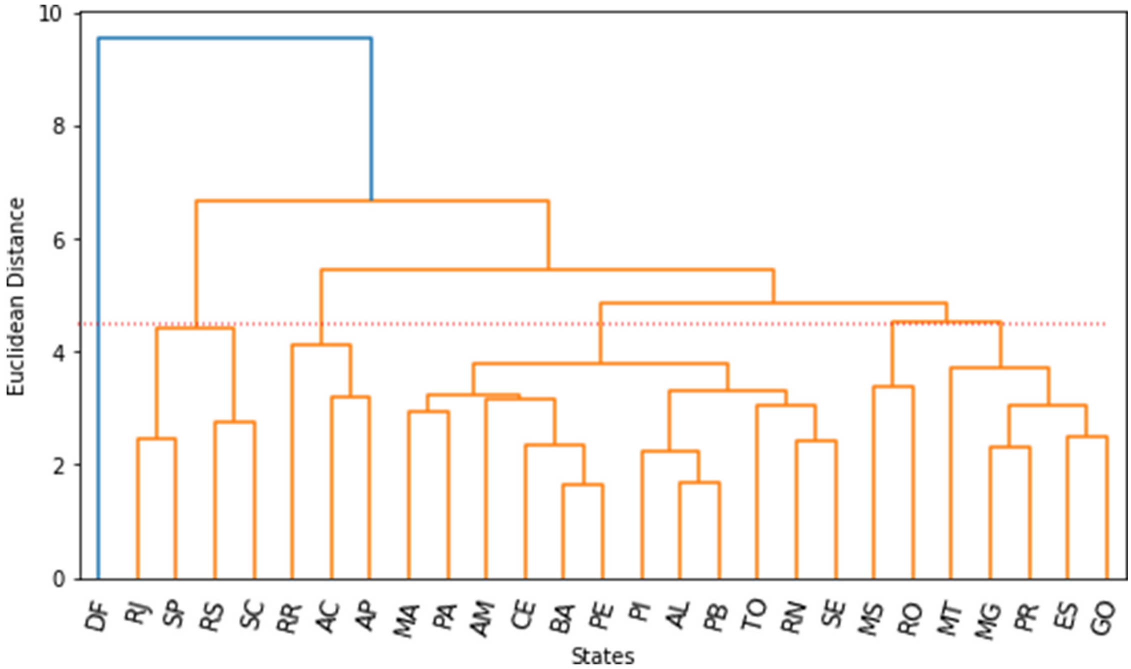

Figure S12. Dendrogram – 4 PQ - Second Scenario.

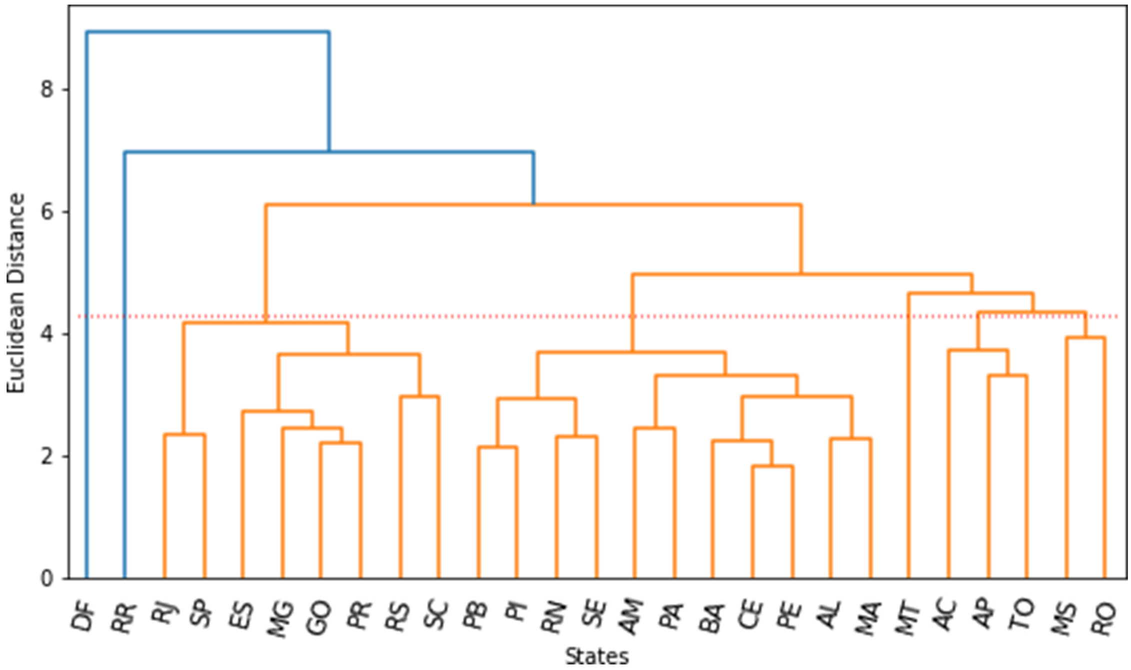

Figure S13. Dendrogram – 5 PQ - Second Scenario.

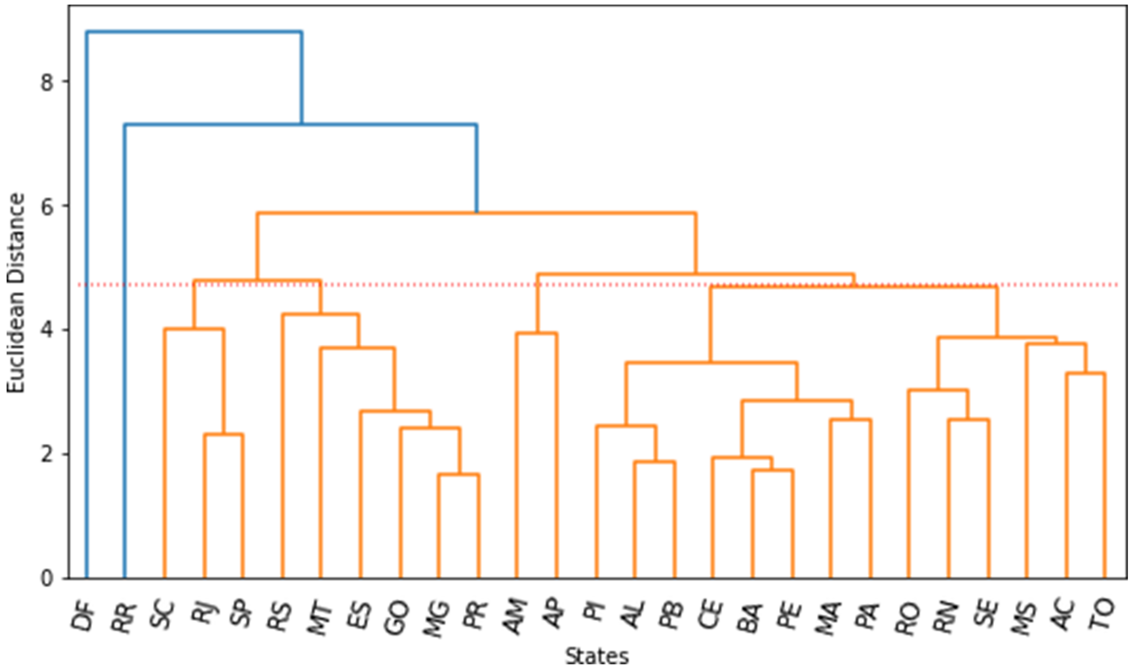

Figure S14. Dendrogram – 6 PQ - Second Scenario.

Third Scenario – Infodemic and Public Influences

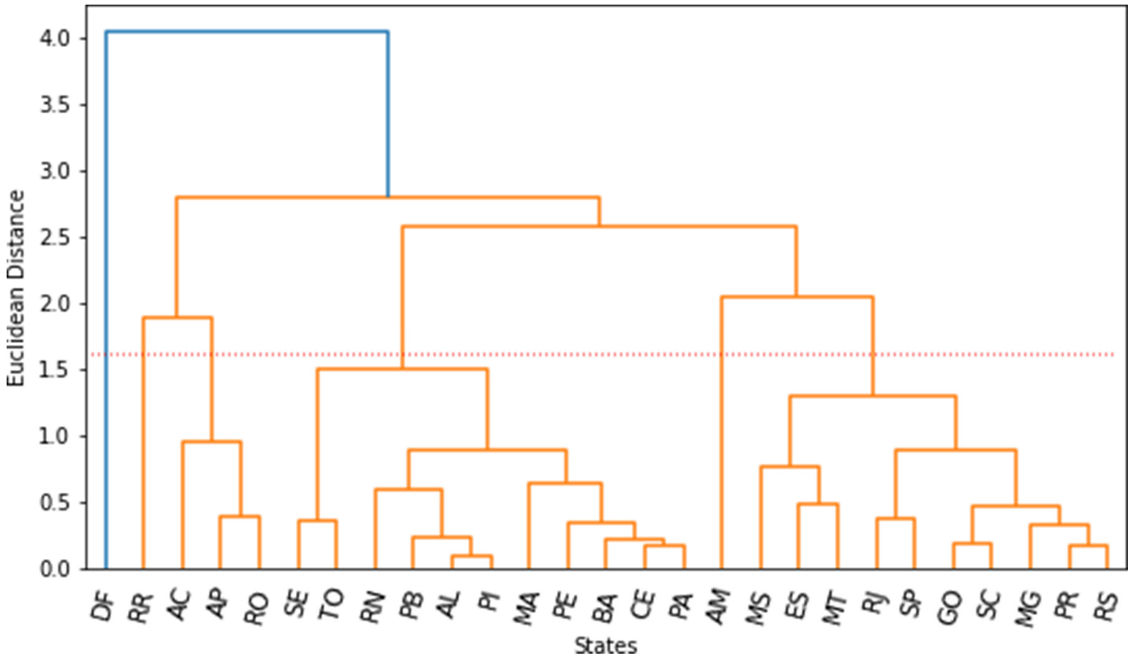

Figure S15. Dendrogram - Third Scenario.
